# Host genotype and age shape the microbial community in the rhizosphere soils of *Camellia* forests

**DOI:** 10.3389/fmicb.2024.1440255

**Published:** 2024-10-01

**Authors:** Jiayan Lv, Chunyu Huo, Jianlang Zhang, Yongfang Huang, Yu Su, Yuzhou Lv, Xianan Xie, Zujing Chen

**Affiliations:** ^1^Guangdong Key Laboratory for Innovative Development and Utilization of Forest Plant Germplasm, College of Forestry and Landscape Architecture, South China Agricultural University, Guangzhou, China; ^2^Guangzhou Collaborative Innovation Center on Science Tech of Ecology and Landscape, Guangzhou Institute of Forestry and Landscape Architecture, Guangzhou, China; ^3^Xiaokeng Forest Farm, Shaoguan, China; ^4^State Key Laboratory of Conservation and Utilization of Subtropical Agro-Bioresources, Guangdong Laboratory for Lingnan Modern Agriculture, South China Agricultural University, Guangzhou, China

**Keywords:** *Camellia oleifera* Abel, forest, genotypes, microbiome, rhizosphere soil

## Abstract

Microbiota living in the rhizosphere influences plant growth and fitness, from the opposite perspective; whether host genotypes control its root microbiota is of great interest to forest breeders and microbiologists. To improve low-yield plantations and promote sustainable management of *Camellia oleifera,* high-throughput sequencing was used to study the chemical properties and microbiome in rhizosphere soil of *Camellia* forests under three genotypes (common *C. oleifera*, local *C. gauchowensis,* and *C. chekiangoleosa*) and three growth stages (sapling stage at 4-year-old, primary fruit stage at 7-year-old, and full fruiting stage at 11-year-old). The results showed that the rhizosphere soil organic matter (OM), nutrient concentrations, diversity, and community composition of the microbiome were significantly varied among different *Camellia* genotypes. The relative abundance of symbiotic and pathotrophic fungi in the rhizosphere soil of *C. chekiangoleosa* was significantly higher than that of *C. gauchowensis*. Concentrations of OM, available phosphorus (AP), and bacterial alpha diversity increased with tree age. Fungi of *Saitozyma*, *Mortierella,* and *Glomeromycota* and bacteria of *Burkholderia–Caballeronia–Paraburkholderia* and *Vicinamibacterales* had potential for fertilizer development for *Camellia* plantation. *Camellia* genotypes and growth stages were significantly correlated with the rhizosphere soil pH, OM, and available potassium (AK). Soil pH and OM were key factors that affected the microbiome in the *Camellia* rhizosphere soils. In conclusion, tree genotypes and growth stages shaped microbial communities in *Camellia* rhizosphere soils, and some plant growth-promoting rhizobacteria were identified as preliminary candidates for improving *Camellia* plantation growth.

## 1 Introduction

Soil supports plant growth and health and is colonized by a large number of microorganisms (Tan et al., 2017). Many studies have shown that soil fertility, pH value, and other soil factors have important effects on the composition and distribution of microbial communities (Guo and Gong, 2014; Wang et al., 2009). Soil pH affects microorganisms not only by regulating enzyme activity but also by determining the ionization balance in the soil, which limits the availability of nutrients (Wang et al., 2014). Soil water content affects soil carbon and nitrogen cycling, plant survival, and soil microbial community structure and function in the natural ecosystems (Gao et al., 2022). Tending practices such as nitrogen fertilizer reduce microbial biomass, respiration rate, and specific functional microbiomes (Leff et al., 2015). Ammonia oxidizers and mycorrhizal fungi are sensitive to high nitrogen levels in natural environments (Leff et al., 2015). The organic matter in the soil promotes the growth and development of microorganisms (Huang et al., 2020). Therefore, there exists a tight association between soil environmental factors and rhizosphere microbial communities, although there is no agreement on which factor plays a crucial role.

Rhizosphere soil is the volume of soil surrounding plant roots that also supports a diverse range of microorganisms. It is influenced by rhizosphere activities and is closely connected to the plant genotypes and rhizosphere secretions (Liu et al., 2019; Wang et al., 2017). The composition of root exudates exhibits significant variation, both across different plants and throughout distinct phases of plant growth (McLaughlin et al., 2023). For instance, *Arabidopsis* secretes sugars and sugar alcohols early on but decreases with plant development, whereas amino acids and phenols increase (Chaparro et al., 2013). Moreover, the unique amino acid profiles in the root secretions from various plants selectively enhance the proliferation of microorganisms with particular biosynthetic capabilities (Cangioli et al., 2022).

Soil fungi and bacteria produce organic matter, sequester carbon, mineralize nutrients, and perform other reactions to transform materials and improve soil chemical properties (Thakur and Geisen, 2019). Therefore, the microbial community structure acts as one of the indicators to evaluate soil fertility and health status (Ochoa-Hueso et al., 2018). Rhizosphere soil produces a unique microclimate that is essential for plant growth and development because the number and activity of rhizosphere microorganisms vary with variety and developmental stage (Hartmann et al., 2008). For example, rhizosphere bacteria can produce phytohormones, volatile organic compounds, biological nitrogen fixation, and quorum-sensing signal interference to promote plant growth (Bhattacharyya and Jha, 2012). Numerous bacteria, including Burkholderia and Bacillus, support plants by inhibiting pathogenic invasions and facilitating the uptake of nutrients from the soil (Deng et al., 2023; Ongena and Jacques, 2008). Moreover, some soil-borne fungi, such as arbuscular mycorrhizal (AM) fungi, can form AM symbiosis with the host plants to promote nutrient and water uptake (Martinez-Garcia et al., 2015). In addition, some rhizosphere fungi, such as Basidiomycota, can also act as decomposers, promoting biogeochemical cycling (Li et al., 2021).

As one of the four woody oil plants cultivated, *Camellia* is widely distributed and cultivated in central and southern China (Gao et al., 2010; Luan et al., 2020). *Camellia* oil extracted from *Camellia* seeds has the excellent characteristics of low cholesterol content and high unsaturated fatty acid content (>90%), which is called “oriental olive oil” and is considered a promising alternative feedstock for biodiesel production (Gao et al., 2015; Yang et al., 2016; Demirbas and Kinsara, 2017). *Camellia oleifera* is generally a perennial evergreen shrub or small tree. Most *Camellia* trees begin to bear fruit at 3–4 years of age, with the fruiting increasing significantly at 6–10 years. After 10 years, they enter a stable and abundant fruiting period. In 2020, the planting area of *Camellia* in China exceeded 4.53 million hectares, the output of *Camellia* oil reached 627,000 tons, and the output value of the *Camellia* oil industry reached 18 billion United States dollars (Chen et al., 2021). According to the data, the area of *Camellia* forestland in Guangdong province was approximately 173,000 ha, accounting for 3.8% of the country’s planted area, and only 24,300 ha of the low-efficiency forestland has been reconstructed. As of December 2021, the national high-yield *Camellia* forestland in China covered only 930,000 hm^2^, representing 21% of the total planted area. Due to improper management and a lack of scientific guidance, the *Camellia* industry is faced with the problems of a low utilization rate of good varieties, a low degree of intensification, scattered management, poor quality of forest land, and low quality of oil tea (Liu et al., 2018). Therefore, it is necessary to investigate the soil health status and rhizosphere microbial characteristics of *Camellia* forestland. This is important for the sustainable development of oil tea forest production and the expansion of the industry.

Recently, several studies have shown that the composition and function of rhizosphere microbial communities are highly dependent on the host genotype, age, and living environments (Martinez-Garcia et al., 2015; Liu et al., 2022; Santolamazza-Carbone et al., 2023). It is known that development phases affect the rhizosphere microbial community features (Hartmann et al., 2008; Kong et al., 2020; Sohn et al., 2021), but there are few studies focusing on the economic forest *Camellia*. *C. oleifera, C. gauchowensis,* and *C. chekiangoleosa* are three species of Camellia, which exhibit significant genetic differences (Dong et al., 2022). In this study, the common *C. oleifera*, local *C. gauchowensis,* and *C. chekiangoleosa* were selected as the main objectives, and the *Camellia* sample plots with the sapling, primary fruit, and full fruiting stages were investigated. Compared with the existing research results, our study made a comparison of the community characteristics of fungi and bacteria in the rhizosphere soil of *Camellia* of three varieties and at three growth stages. The sensitivity of the microbial community to genotypes and ages was further analyzed. This study is of great significance for the sustainable management of *Camellia* plantations by screening out potential beneficial microorganisms.

The aim of our study was to find out the effects of genotypes and ages on bacteria and fungi in the rhizosphere soil of *Camellia* forestland. As we suspected, plant genotypes and growth stages regulate microbial diversity in rhizosphere soils. There were significant differences in soil organic matter, nutrient concentrations, microbial community diversity, and composition in rhizosphere soil of different genotypes. The concentration of some nutrients in the soil also changes with age. Since the samples were collected from the same tree farm and managed in the same way, we hypothesized that these differences are closely related to plant genotypes and growth stages.

## 2 Materials and methods

### 2.1 Study sites and sample collection

Study sites were located at the state-owned Xiaokeng Forest Farm (24° 15’ N, 113° 35′ E) in Qujiang District, Shaoguan City, Guangdong Province. The climate type of study sites was subtropical monsoon climate. The terrain was mainly mountainous and hilly. The average annual temperature was 20.3°C, the average annual rainfall was 1,530 mm, and the annual sunshine duration was 1706 h. The extreme maximum and the lowest temperatures were 40.2°C and − 5.3°C, respectively. The region receives the highest amount of rainfall from April to June every year. The frost descent period was approximately 15 days every year. The soil type was acid red soil with a thickness of more than 1 m. The soil moisture content in this forest was 23.11%, with a pH of 4.79 and an organic matter content of 12.68 g kg^−1^. The concentrations of total nitrogen, total phosphorus, total potassium, available nitrogen, available phosphorus, and available potassium were 0.76 g kg^−1^, 0.31 g kg^−1^, 18.96 mg kg^−1^, 58.56 mg kg^−1^, 0.74 mg kg^−1^, and 50.17 mg kg^−1^, respectively. The soil is classified as acidic.

*Camellia* forests used the horizontal terraced land preparation method, the bandwidth was between 2 and 3 m, and the hole size was 0.5 m × 0.5 m × 0.4 m. The *Camellia* trees were spaced at 2.5 m x 3.0 m. Loosening and weeding were done twice a year, in the summer and fall of the year. The basal fertilizer was applied during the hole-digging process, with 0.25 kg of phosphorus fertilizer applied per hole. Fertilizer was applied twice a year, in the spring with fast-acting fertilizer, urea 0.5 kg per plant, and in the winter with slow-release fertilizer, 2 kg per plant. *Camellia* of the same species but of different ages is grown in adjacent areas, and different species are grown separately.

Rhizosphere soil was collected on 1 July 2022. The rhizosphere soils were collected from three different genotypes of *Camellia* spp.: common *C. oleifera*, local *C. gauchowensis,* and *C. chekiangoleosa* at 11-year-old, and at three age groups of common *C. oleifera*: sapling stage at 4-year-old, primary fruit stage at 7-year-old, and full fruiting stage at 11-year-old as shown in Supplementary Table S1.

The healthy *Camellia* with consistent growth status were randomly selected on the study sites. A total of 50 rhizosphere soil samples were collected from 5 different *Camellia* plots, with 10 samples taken from each plot. Soil from two plants in the same plot was randomly combined into one sample, resulting in 5 samples per plot. Therefore, a total of 25 soil samples were used for the experiment. Samples were collected from approximately 1 m away from the trunk (He et al., 2021). According to He et al. (2021) 5–20 cm length of roots with a diameter of less than 1 mm were collected, and the soil not closely attached to the roots was removed by gently shaking. The soil attached to these *Camellia* roots approximately 1 mm thick was considered as the rhizosphere soil. The rhizosphere soil was collected and put into the sterile sampling bags, then placed in dry ice, and brought back to the laboratory. The root and soil samples were transferred to a 50 mL sterile centrifuge tube containing 20 mL sterile phosphate-buffered saline (PBS) buffer and placed on a full-temperature oscillator. The samples were oscillated for 20 min at room temperature at 120 rpm/min. The roots were picked out into a 50 mL centrifuge tube with sterile tweezers, and the remaining suspensions were centrifuged at a high speed (6,000 × g, 4°C) for 20 min to collect the rhizosphere soil. The rhizosphere soil samples were divided into two parts: one was stored at −80°C for the extraction of soil total DNA, and the other was used for the determination of soil chemical properties. The soil’s chemical properties were measured using 30 g of 2 mm screened soil.

### 2.2 Soil physicochemical property analysis

The pH value, water content (WC), organic matter (OM), available phosphorus (AP), total nitrogen (TN), alkali-hydrolyzed nitrogen (AN), and available potassium (AK) of the rhizosphere soil samples were essentially determined as the following: The pH value and moisture content of soil were determined by the glass electrode method and the drying method, respectively (Liu et al., 2017; Yin et al., 2021). Organic matter content was determined by high temperature external thermal potassium dichromate oxidation-volumetric method (Dong et al., 2018). The content of available phosphorus was determined by the molybdenum-antimony colorimetric method extracted from hydrochloric acid and ammonium fluoride (Yin et al., 2021). The total nitrogen content was determined by Kelvin-distillation titration (Liu et al., 2017). Alkali-hydrolyzed nitrogen content was determined by alkali-diffusion method, and available potassium content was determined by ammonium acetate extraction and flame atomic absorption spectrophotometry (Cui et al., 2018).

### 2.3 DNA library construction and sequencing

Total soil DNA was extracted by the TGuide S96 magnetic soil DNA kit (DP812, TIANGEN); then, DNA purity/impurity and concentration were detected by the agarose gel electrophoresis (Bomei Fuxin Technology Co. Ltd., Beijing, China) and microplate reader (synergy HTX, Gene Company Limited), respectively. After DNA extraction, the bacterial 16S rDNA V3-V4 region (338F, 5’-ACTCCTACGGGAGGCAGCA-3′, 806R, 5’-GGACTACHVGGGTTWTCTAAT-3′) and the fungal ITS region (ITS1-F, 5’-CTTGGTTCATTTAGAGGAAGTAA-3′; ITS2R, 5’-GCTGCGTTCTTCATCGATGC-3′) were amplified for obtaining the target 16S and ITS DNA fragments to prepare the amplicon libraries, and the DNA pools were sent to the Beijing Baimaike Biotechnology Co., Ltd. for Illumina NovaSeq 6,000 sequencing.

### 2.4 Data analysis

The raw reads obtained by Illumina sequencing were filtered using Trimmomatic V 0.33 software. Clean reads were obtained by identifying and removing primer sequences using the Cutadapt 1.9.1 software. USEARCH V 10 software was used to overlap and splice clean reads of each sample, and the length of the spliced data was screened according to the length range of different regions. The dada2 method in QIIME2 2020.6 software was used to denoise and remove chimeric sequences to obtain the final effective data (non-chimeric reads). The species taxonomic annotation of the bacterial and fungal amplicon sequence variants (ASVs) was based on the SILVA (V. 138) rRNA gene database and UNITE (V. 8.0), respectively. The conservative threshold for ASV filtration was 0.005%. The feature sequences were taxonomically annotated using Naive Bayes Classifiers with a confidence interval set to 0.7. (1) Trimmomatic parameter setting: Window size was set as 50 bp. The reads were cut from the start of the window once the average Q-score within the window was lower than 20. (2) Primer identification and removal: Cutadapt was applied to remove the primer sequences. Parameter setting: Maximum mismatch accepted: 20%; minimum coverage: 80%. (3) PE reads assembly: PE reads were assembled by Usearch v10. Parameter setting: Minimum length of overlap: 10 bp; minimum similarity within the overlapping region: 90%; maximum mismatch accepted: 5 bp (Default). (4). Each query sequence was split into non-overlapped chunks. These chunks were compared with the reference database to identify the best hit of each chunk in the database and further define the two best parent sequences. The query sequence was subsequently compared with the two parent sequences. If a fragment with over 80% similarity to the query sequence is found on both parents, this query sequence will be defined as a chimera sequence. A total of 1,978,367 clean reads were obtained from the rhizosphere soil samples of three *Camellia* genotypes under five plots based on the fungal ITS region sequencing. At least 73,474 clean reads were generated in each sample, with an average of 79,135 clean reads. On the other hand, a total of 1,995,450 clean reads were obtained from the rhizosphere soil samples of three *Camellia* genotypes under five plots based on the bacterial 16S sequencing. At least 79,337 clean reads were generated in each sample, with an average of 79,818 clean reads. The analysis of sequencing data was based on Baimai Cloud platform (www.biocloud.net).

Statistically significant differences in chemical properties and alpha diversity indices of soils were analyzed using SPSS 19.0 software (IBM Corp, Armonk, NY). Duncan’s test was used for the difference and significance analyses. Based on the R language platform (v3.1.1) and Bray–Curtis algorithm, the non-metric multidimensional scaling (NMDS) analysis and redundancy analysis (RDA) were established. Species abundance histograms were established using python2 (matplotlib-v1.5.1) (R Core Team, 2021). The FUNGuild annotation tool was used to predict the distribution of fungal nutrient patterns (Nguyen et al., 2016). Gephi V 0.10.1 was used for the symbiotic network’s visualization analysis, and the network’s topological features were calculated (Bastian et al., 2009). Based on the unweighted UniFrac and Bray–Curtis algorithm, the analysis of similarities (ANOSIM) was performed using the vegan package in R language, and boxplots were drawn using Python. TBtools (v1.09) was used to draw the Venn diagram (GitHub, 2021).

## 3 Results

### 3.1 The chemical properties of the rhizosphere soil of *Camellia* vary with different genotypes and growth stages

The chemical properties of the rhizosphere soils of three *Camellia* genotypes were significantly affected by the host genotypes and growth stages (Table 1). There were significant differences in the organic matter (OM), total nitrogen (TN), available phosphorus (AP), available potassium (AK), and water content (WC) in the rhizosphere soils of different genotypes. The rhizosphere soils of five plots were acidic, with a pH range from 4.54 to 5.56. The rhizosphere soil pH value of C11 (5.56) was significantly higher than that of H11 (4.56) and G11 (4.54). The pH value was increased from 4.6 to 5.56 at planting years from sapling stage to full fruiting stage in the rhizosphere soils of *C. oleifera;* this indicated that the rhizosphere soil pH may be mediated by the plantation of *C. oleifera*. Among the different genotypes, the concentrations of OM, TN, AN, and WC in the rhizosphere soils of H11 were the highest, while the content of AP was the lowest (Table 1). By contrast, the concentrations of OM, TN, and WC in the rhizosphere soils of G11 (age 11) were the lowest, but the content of AP was the highest. Compared with different age groups, the concentrations of OM, TN, AN, AP, AK, and WC and pH values in the rhizosphere soil of *C. oleifera* at the sapling stage were the lowest (Table 1) and significantly increased in the rhizosphere soil of *C. oleifera* at the primary fruit stage. The concentrations of TN, AN, AK, and WC in the rhizosphere soil of *C. oleifera* were the highest at the primary fruit stage but decreased significantly at the full fruiting stage. The concentrations of OM and AP in the rhizosphere soils increased with the growth stages of *C. oleifera*.

**Table 1:** Physicochemical properties in the rhizosphere soils of *C. oleifera*.

Sample	pH	OM (g/kg)	TN (g/kg)	AN (mg/kg)	AP (mg/kg)	AK (mg/kg)	WC (%)
C4	4.6 ± 0.01c	13.48 ± 0.16d	0.57 ± 0e	47.04 ± 1.68d	4.97 ± 0.33c	53.71 ± 1.81d	24.88 ± 0.33e
C7	5.4 ± 0.08b	21.9 ± 0.13c	1.24 ± 0.01b	114.24 ± 1.68b	5.63 ± 0.06c	188.05 ± 2.49a	34.25 ± 0.19b
C11	5.56 ± 0.02a	24.06 ± 0.39b	1.13 ± 0.02c	92.4 ± 1.68c	28.85 ± 0.46b	176.66 ± 1.41b	33.2 ± 0.28c
G11	4.54 ± 0.01c	21.64 ± 0.37c	0.99 ± 0.01d	94.42 ± 3.2c	43.14 ± 1.56a	62 ± 0.84c	28.82 ± 0.04d
H11	4.56 ± 0c	29.73 ± 0.15a	1.49 ± 0.03a	147.84 ± 3.36a	0.44 ± 0.03d	42.22 ± 1.1e	35.42 ± 0.03a

^*^OM, organic matter; TN, total nitrogen; TK, total potassium; AN, alkali-hydrolyzed nitrogen; AP, available phosphorus; AK, available potassium; WC, water content. C4, sapling stage of *C. oleifera*; C7, primary fruit stage of *C. oleifera*; C11, full fruiting stage of *C. oleifera*; G11, 11-year-old *C. gauchowensis*; H11, 11-year-old *C. chekiangoleosa*. Data shown in the table indicate the mean ± standard error (SE). Different lowercase letters in the same column indicate significant differences (*p* < 0.05), based on Duncan’s test.

### 3.2 Microbial community diversity in the rhizosphere soils of three *Camellia* genotypes under five plots

**Figure 1:**
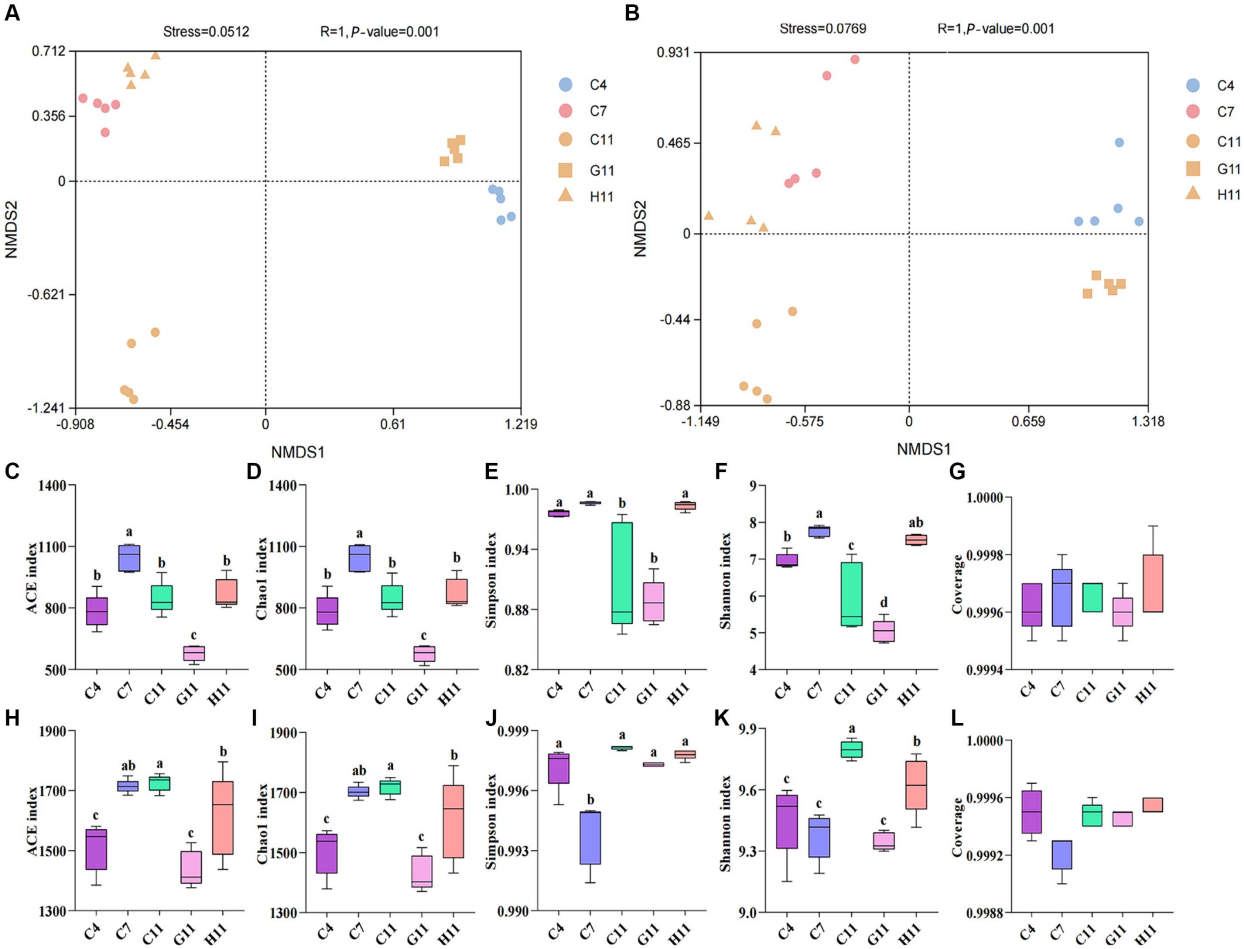
Non-metric dimensional scaling (NMDS) visualization of the beta diversity in the rhizosphere soils of three *Camellia* genotypes under five plots. **(A)** NMDS visualization of the fungi; **(B)** NMDS visualization of the bacteria. *R* and *p*-values are the results of ANOSIM analysis. *p*-value indicates the *p*-value of significance for within-group intergroup comparisons; R indicates the degree of difference between intergroup and within-group comparisons; Generally, *R* > 0.75 means a large difference. Rarefaction curves and alpha diversity indexes of the microbial community in the rhizosphere soils of three *Camellia* genotypes under five plots. **(A)** Rarefaction curves of fungal communities. **(B)** Rarefaction curves of bacterial communities. **(C)** ace index of the fungi; **(D)** chao1 index of the fungi; **(E)** Simpson index of the fungi; **(F)** Shannon index of the fungi; **(G)** coverage of the fungi; **(H)** ace index of bacterial communities; **(I)** Chao1 index of bacterial communities; **(J)** Simpson index of bacterial communities; **(K)** Shannon index of bacterial communities; **(L)** coverage of bacterial communities. C4: sapling stage of *C. oleifera*; C7: primary fruit stage of *C. oleifera*; C11: full fruiting stage of *C. oleifera;* G11: full fruiting stage of *C. gauchowensis*; H11: full fruiting stage of *C. chekiangoleosa.* The five treatments were compared for significance using the Duncan test, and different letters were assigned to indicate these significant differences in individual parameters (*p* < 0.05).

The beta diversity of fungi in the rhizosphere soils of different *Camellia* was similar to that of the bacteria. There are slight differences in the distribution of root soil microorganisms between H11 and C11, but there are significant disparities in the microbial group distribution among H11, C11, and G11 (Supplementary Figures S1A,B). The alpha diversity index of fungi in rhizosphere soils was considerably varied throughout the development phases of *C. oleifera*, with the primary fruit stage being much greater than the sapling and full fruit stages (Figures 1C–F). The alpha diversity index of fungi in the rhizosphere soils varied among different *Camellia* genotypes, with the highest diversity observed in H11, followed by C11 and then G11. The fungal diversity indexes (Simpson and Shannon) of H11 were significantly higher than those of C11 and G11, while the fungal alpha diversity indexes (Ace and Chao1) of G11 were significantly lower than those of H11 and C11. On the other hand, the Ace, Chao1, and Shannon indexes of bacteria in the rhizosphere soils of *Camellia* exhibited significant differences among the genotypes, with the highest indexes observed in C11, followed by H11 and then G11 (Figures 1H–K). The Simpson index of bacteria in the rhizosphere soil of *C. oleifera* showed a trend of initially decreasing and then increasing, and the Ace and Chao1 indexes increased with the growth stages of *C. oleifera*. The alpha diversity of fungi and bacteria was the lowest in the G11 rhizosphere soil.

The rarefaction curves of the soil samples gradually flattened (Figures 1A,B) and the sequencing coverage was between 0.99 and 1.00 (Figures 1G,L), indicating that the sample sequences were sufficient for subsequent data analysis. There is a minor discrepancy in the distribution of soil microorganisms between the primary fruit (C7) and full fruiting stages (C11). However, there are evident variations in the distribution of microorganisms among the primary fruit (C7), full fruiting stages (C11), and the sapling stage (C4). ANOSIM boxplots show that the difference between the microbes in different groups is significant (Supplementary Figures S1C,D).

### 3.3 The rhizosphere soils of *Camellia* exhibit a small overlap of ASVs across different host genotypes and growth stages

**Figure 2:**
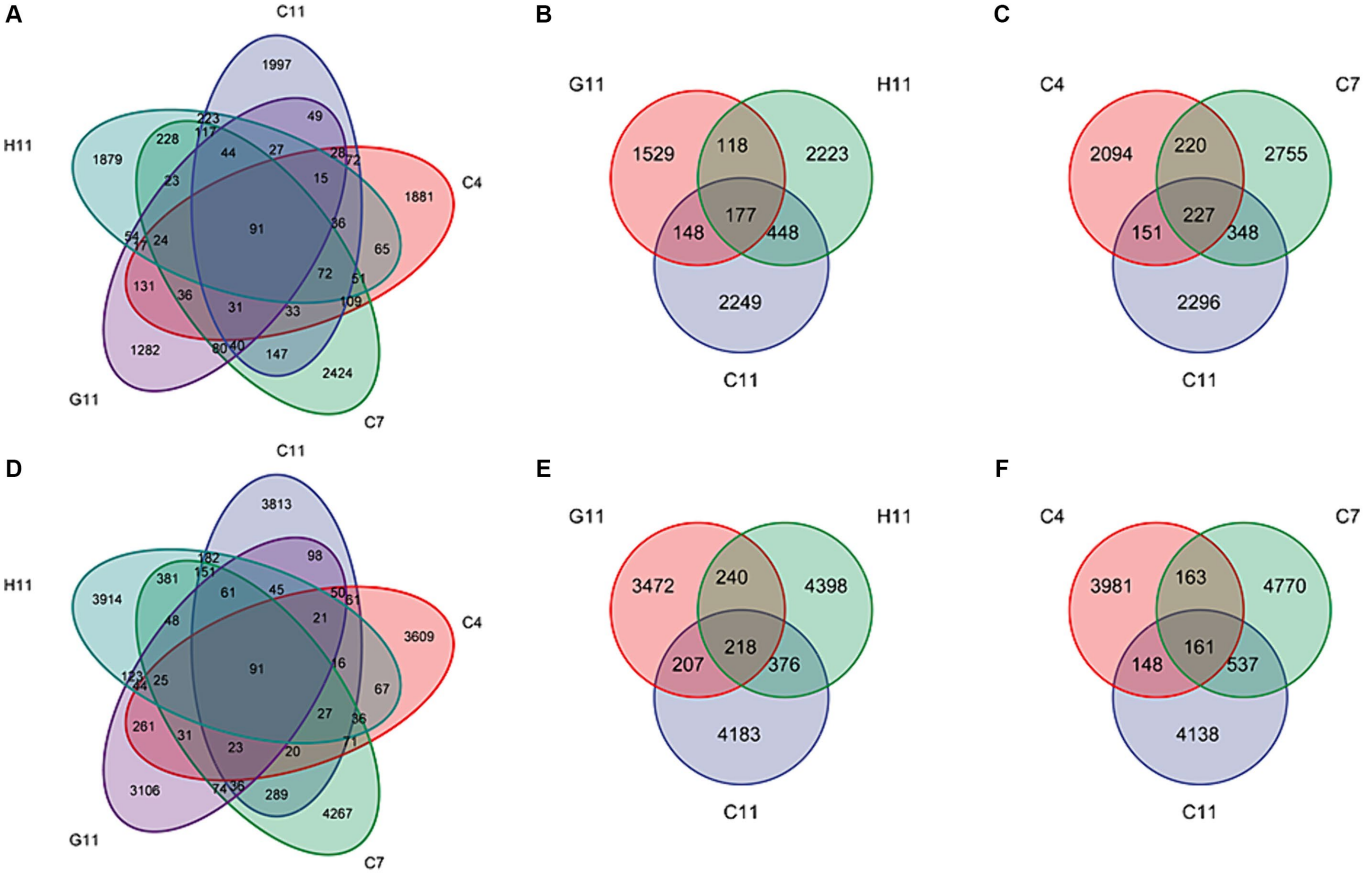
Venn diagrams for the ASV numbers of the microbiota in the rhizosphere soils of three *Camellia* genotypes under five plots. Venn diagram of shared and unique fungi **(A)** and bacteria **(D)** ASVs among five groups. Venn diagram of ASVs of shared and unique fungi **(B)** and bacteria **(E)** among three genotypes. Venn diagram of ASVs of shared and unique fungi **(C)** and bacteria **(F)** among three growth stages. C4: sapling stage of *C. oleifera*; C7: primary fruit stage of *C. oleifera*; C11: full fruiting stage of *C. oleifera*; G11: full fruiting stage of *C. gauchowensis*; H11: full fruiting stage of *C. chekiangoleosa.*

Among the 25 samples of fungal and bacterial community composition in the rhizosphere soils of *C. oleifera*, *C. gauchowensis*, and *C. chekiangoleosa*, a total of only 91 amplicon sequence variants (ASVs) were found to be shared. These overlapping ASVs represented a mere 0.80 and 0.43% of the respective total ASVs for each plant species (Figures 2A,D). The microbiota amplicon sequence variants (ASVs) in the rhizosphere soils of three *Camellia* genotypes exhibited variations across different growth stages and were influenced by the host genotype. In the rhizosphere soils of *Camellia*, there were 177 and 277 overlapping fungal amplicon sequence variants (ASVs) under different host genotypes and growth stages, respectively. These overlapping ASVs accounted for only 2.57 and 2.81% of their corresponding total fungi. In addition, in the C11, G11, and H11 rhizosphere soils, there were 2,249, 1,529, and 2,223 specific fungi ASVs, respectively. These specific ASVs accounted for 32.63, 22.19, and 32.25% of their corresponding total ASVs, respectively (Figure 2B). The specific fungi ASVs in the rhizosphere soils of *C. oleifera* at the sapling, primary fruit, and full fruiting stages were 2,094, 2,755, and 2,296, respectively, which accounted for 25.88, 34.05, and 28.38% of their corresponding total ASVs, respectively (Figure 2C).

There were 218 and 161 overlapping bacterial amplicon sequence variants (ASVs) in the rhizosphere soils of *Camellia* under different host genotypes and growth stages. These overlapping ASVs accounted for only 1.66 and 1.16% of their corresponding total ASVs, respectively. Moreover, in the rhizosphere soils of C11, G11, and H11, there were 4,183, 3,472, and 4,398 specific bacterial ASVs, respectively. These specific ASVs accounted for 31.95, 26.52, and 33.59% of their corresponding total ASVs, respectively. (Figure 2E). The specific bacterial ASVs of *C. oleifera* at the sapling, primary fruit, and full fruiting stages were 3,981, 4,770, and 4,138, respectively, accounting for 28.64, 34.32, and 29.77% of their corresponding total ASVs, respectively (Figure 2F), which were the highest at the primary fruit stage.

The number of ASVs in the rhizosphere soils of C11 and H11 was higher than that in G11 (Supplementary Figure S2). As the growth stage of *C. oleifera* increased, the number of specific ASVs of fungi and bacteria initially peaked at the primary fruit stage and then decreased. The variation range of fungi in the rhizosphere soils of three *Camellia* genotypes under five plots was found to be higher than that of bacteria. Overall, the ASVs in the rhizosphere soils of *Camellia* showed a limited overlap (1.16–2.81%) across different host genotypes and growth stages.

### 3.4 The relative abundance of dominant phyla and dominant genera of fungi and bacteria in the rhizomatous soil of different *Camellia* genotypes varied considerably

**Figure 3:**
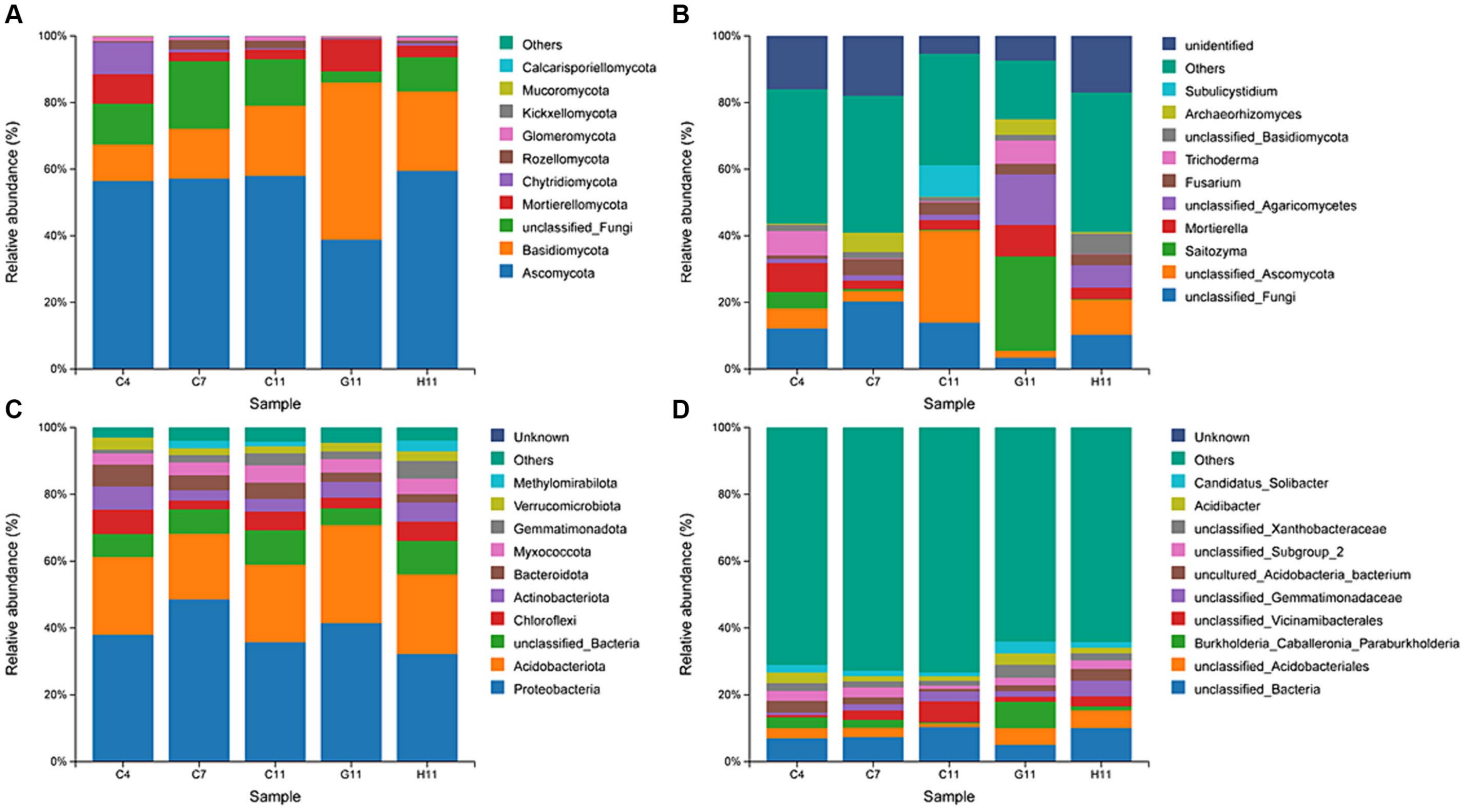
Relative abundances of fungi and bacteria at phyla and genus levels. **(A,B)** Relative abundances of fungi at phyla and genus levels. **(C,D)** Relative abundance of bacteria at phyla and genus levels. C4: sapling stage of *C. oleifera*; C7: primary fruit stage of *C. oleifera*; C11: full fruiting stage of *C. oleifera*; G11: full fruiting stage of *C. gauchowensis*; H11: full fruiting stage of *C. chekiangoleosa*.

The dominant phyla of fungi in the rhizosphere soil were Ascomycota (38.81–59.47%) and Basidiomycota (11.04–47.18%), accounting for over 67% of the relative abundance (Figure 3A). The dominant genera of fungi in the rhizosphere soil of the three *Camellia* genotypes across five plots included *Saitozyma* (0.20–28.31%), *Mortierella* (2.52–9.46%), *Fusarium* (1.02–4.83%), and *Trichoderma* (0.13–7.32%) (Figure 3B). The relative abundance of dominant fungal phyla and genera in the rhizosphere soil of different *Camellia* genotypes exhibited significant variations. In terms of dominant fungal phyla, the relative abundance of Ascomycota and Glomeromycotina in G11 was notably lower compared to C11 and H11. Conversely, the relative abundance of Basidiomycota and Mortierellomycota was significantly higher in G11 than in C11 and H11. The relative abundance of *Saitozyma*, *Mortierella*, *Trichoderma*, and *Archaeorhizomyces* showed a significant increase in G11 than in C11 and H11.

The dominant phyla of bacteria in the rhizosphere soils of the three *Camellia* genotypes across five plots were Proteobacteria (32.21–48.58%) and Acidobacteriota (19.60–29.38%), accounting for more than 56% of the relative abundance (Figure 3C). The dominant genera of rhizosphere soil bacteria included an unclassified genus of *Acidobacteriales* (1.12–5.31%) and *Burkholderia–Caballeronia–Paraburkholderia* (0.37–7.86%) (Figure 3D). At the level of the dominant bacterial phylum, the relative abundance of Proteobacteria and Acidobacteriota was significantly higher in G11 than in C11 and H11. The relative abundance of Chloroflexi, Myxococcota, Gemmatimonadota, and Methylomirabilota in G11 was significantly lower than that in C11 and H11. At the level of dominant bacterial genera, the relative abundance of *Burkholderia–Caballeronia–Paraburkholderia*, *Acidibacter*, and *Candidatus Solibacter* was significantly higher in G11 than in C11 and H11.

### 3.5 Fungal trophic groups in the rhizosphere soils of three *Camellia* genotypes under five plots

**Figure 4:**
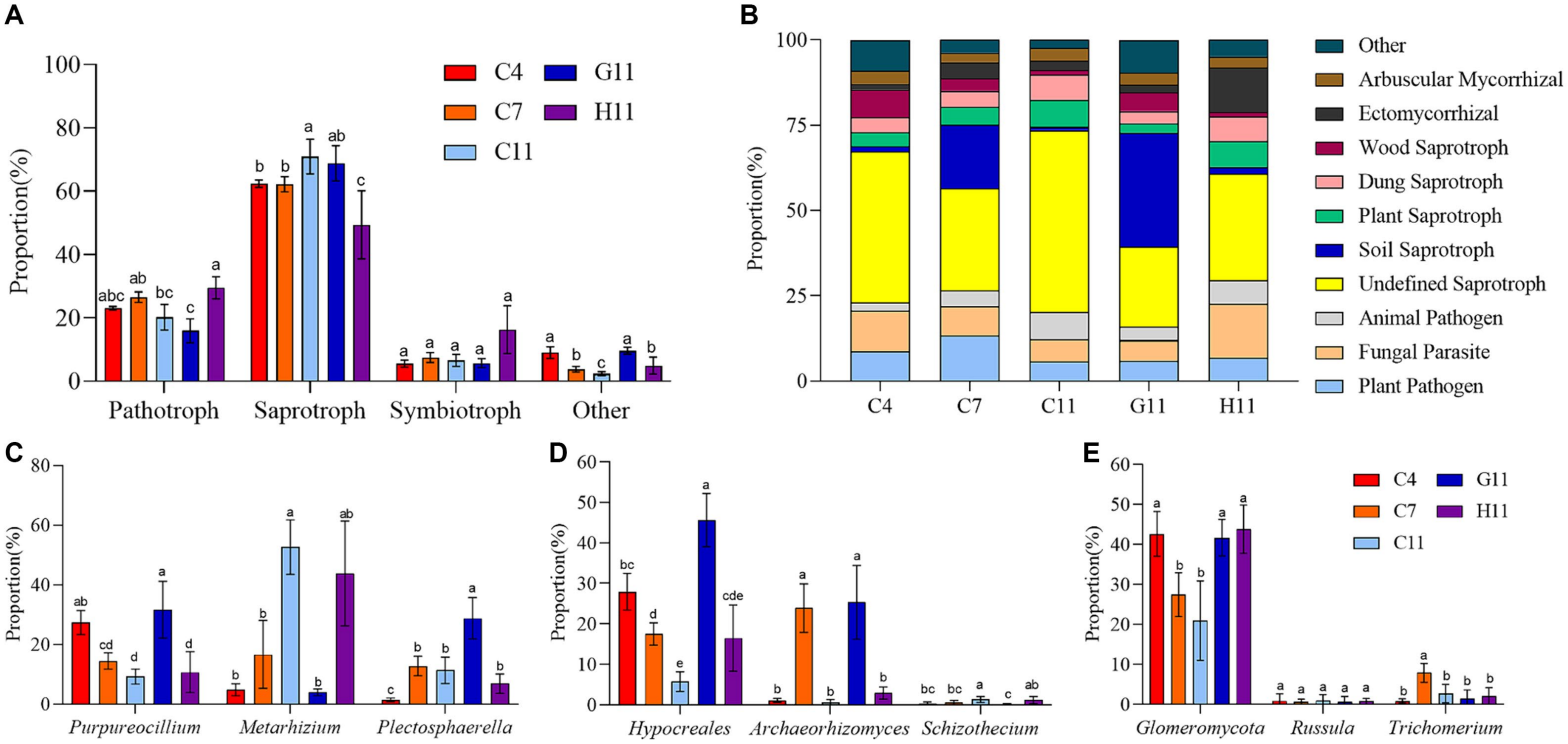
Fungal trophic groups in the rhizosphere soils of three *Camellia* genotypes under five plots. **(A)** Variations of fungal functional; **(B)** composition of fungal function group. **(C–E)** The fungi with the largest proportion among the major trophic functional groups. Different letters indicate significant differences in individual parameters among five plots (*p* < 0.05). C4: sapling stage of *C. oleifera*; C7: primary fruit stage of *C. oleifera*; C11: full fruiting stage of *C. oleifera*; G11: full fruiting stage of *C. gauchowensis*; H11: full fruiting stage of *C. chekiangoleosa*. The five treatments were compared for significance using the Duncan test, and different letters were assigned to indicate these significant differences in individual parameters (*p* < 0.05).

In the rhizosphere soils of three *Camellia* genotypes across five plots, the relative abundance of pathotrophic, saprotrophic, and symbiotrophic groups ranged from 16 to 29%, 62 to 71%, and 6 to 16%, respectively The relative abundance of the pathotrophic, saprotrophic, and symbiotrophic groups in the rhizosphere soils of three *Camellia* genotypes under five plots were 16–29%, 62–71%, and 6–16%, respectively (Figure 4A). The 10 main nutrient function groups in the rhizosphere soil of three *Camellia* genotypes under five plots were plant pathogens, fungal parasites, animal pathogens, undefined saprotrophs, soil saprotrophs, plant saprotrophs, dung saprotrophs, wood saprotrophs, ectomycorrhizal (ECM), and AM fungi (Figure 4B).

In the rhizosphere soil of three *Camellia* genotypes under five plots, the main pathotrophic fungi were *Purpureocillium* and *Metarhizium* (Figure 4C). The proportion of *Purpureocillium* significantly decreased with the growth stage and was significantly higher in G11 than in C11 and H11. On the contrary, the proportion of *Metarhizium* significantly increased with the growth stage and also was significantly higher in C11 than in G11. The main saprotrophic groups of fungi were *Hypocreales* (undefined saprotroph), *Archaeorhizomyce* (soil saprotroph), and *Schizothecium* (dung saprotroph), and they were significantly affected by the growth stages of *Camellia* (Figure 4D). The abundance of *Hypocreales* decreased with plant age, showing significant variations among growth stages. *Archaeorhizomyce* had the highest proportion in the rhizosphere soil during the primary fruit stage, which was significantly greater than during the sapling and full fruiting stages. In contrast, *Schizothecium* had a significantly higher relative abundance in the rhizosphere soil during the full fruiting stage compared to the sapling and primary fruit stages. In addition, the proportions of *Hypocreales* and *Archaeorhizomyce* were significantly higher in G11 than in C11 and H11, but the proportion of Schizothecium in the rhizosphere soil of G11 was significantly lower than in C11 and H11. The symbiotrophic fungi in the rhizosphere soils of three *Camellia* genotypes under five plots were mainly composed of AM fungi and ECM fungi. The relatively large proportion of AM fungi, ctomycorrhizae, and endophytic fungi were Glomeromycotina, *Russula,* and *Trichomerium*, respectively (Figure 4E). The proportion of Glomeromycotina in G11 and H11 was significantly higher than that in C11. The proportion of Glomeromycota at the sapling stage was significantly higher than at the full fruiting stage.

### 3.6 Soil pH, OM, AP, and AK were significantly affected by host genotypes and growth stages

**Figure 5:**
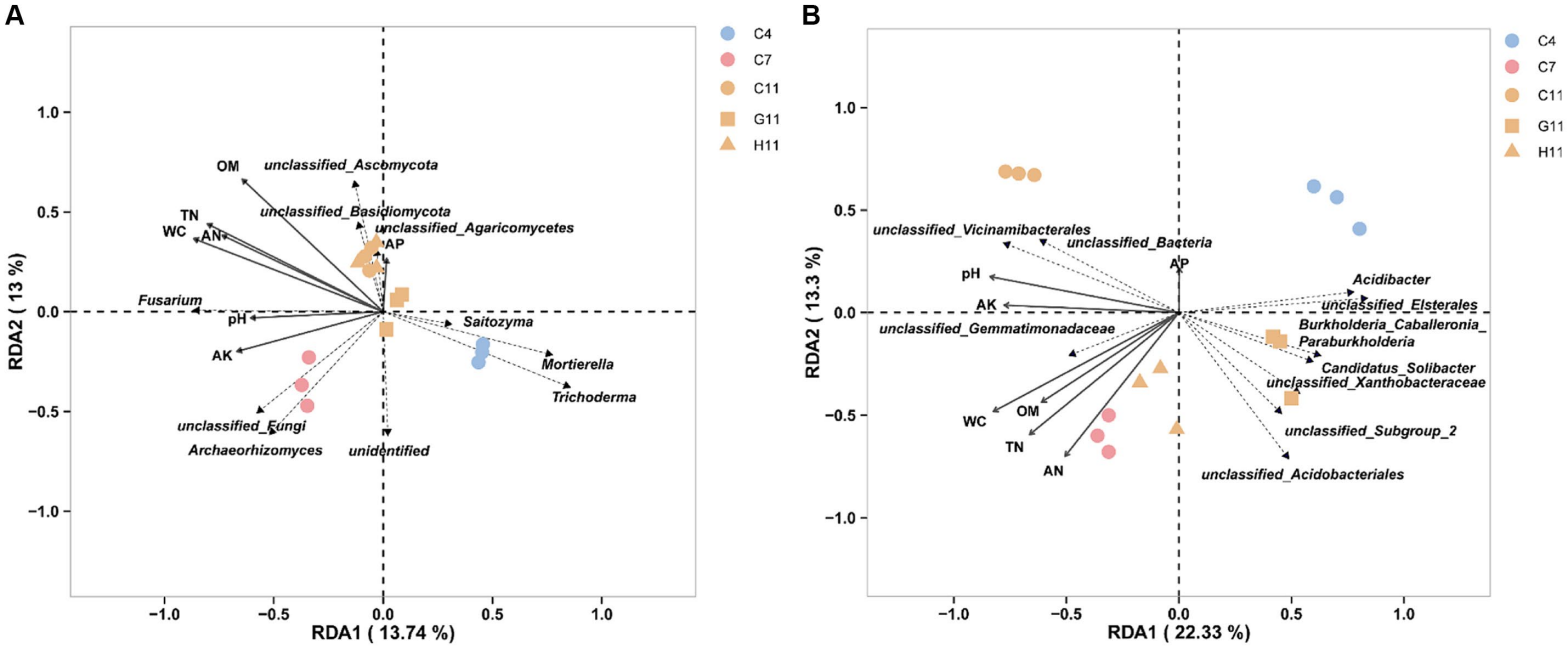
Bacterial function analysis and RDA. **(A)** The RDA of the top 10 dominant fungal taxa and soil environmental factors in the rhizosphere soils of three *Camellia* genotypes under five plots at the genus level. **(B)** The RDA of the top 10 dominant bacterial taxa and soil environmental factors in the rhizosphere soils of three *Camellia* genotypes under five plots at the genus level. The length of the arrow represents the degree of association with fungi. The acute angle, obtuse angle, and right angle between the arrows represent the positive, negative, and no correlation, respectively. RDA can display samples and environmental factors on the same two-dimensional ordination plot, allowing for a visual representation of the relationship between sample distribution and environmental factors. The dotted line in the figure labels microbial genera, and the solid line represents environmental factors. C4: sapling stage of *C. oleifera*; C7: primary fruit stage of *C. oleifera*; C11: full fruiting stage of *C. oleifera*; G11: full fruiting stage of *C. gauchowensis*; H11: full fruiting stage of *C. chekiangoleosa*.

The RDA of the top 10 fungal genera and soil environmental factors revealed that the ranking axis accounted for 26.74% of the community variation in the rhizosphere soil samples of three *Camellia* genotypes across five plots (Figure 5A). The key soil environmental factors correlated to fungal communities were WC (*r*^2^ = 0.86, *p* = 0.001), OM (*r*^2^ = 0.83, *p* = 0.001), TN (*r*^2^ = 0.82, *p* = 0.001), AN (*r*^2^ = 0.67, *p* = 0.001), and AK (*r*^2^ = 0.48, *p* = 0.023) (Supplementary Table S2). The results demonstrated that *Archaeorhizomyces* showed a positive correlation with the concentrations of WC, TN, AK, AN, and pH value in the rhizosphere soils, while *Saitozyma*, *Mortierella*, and *Trichoderma* exhibited a negative correlation. In addition, *Fusarium* showed a positive correlation with the concentrations of WC, TN, AK, AN, pH, OM, and pH values. Similarly, the RDA of the top 10 bacterial genera and soil environmental factors showed that the ranking axis explained 35.63% of the community variation in the rhizosphere soil samples of three *Camellia* genotypes under five plots (Figure 5B). The key environmental factors affecting bacterial communities were WC (*r*^2^ = 0.91, *p* = 0.001), TN (*r*^2^ = 0.79, *p* = 0.001), AN (*r*^2^ = 0.74, *p* = 0.001), pH (*r*^2^ = 0.73, *p* = 0.002), AK (*r*^2^ = 0.60, *p* = 0.007), and OM (*r*^2^ = 0.56, *p* = 0.01) (Supplementary Table S2). The results indicated a positive correlation between *Gemmatimonadetes* and *Vicinamibacterales* with the concentrations of WC, TN, AK, AN, OM, and pH value, while *Burkholderia–Caballeronia–Paraburkholderia*, *Xanthobacteraceae*, *Candidatus Solibacter*, *Elsterales*, and *Acidibacter* showed a negative correlation with the concentrations of these soil environmental factors. *Acidobacteriales*, on the other hand, exhibited a positive correlation with AN content but a negative correlation with the other mentioned environmental factors.

**Figure 6:**
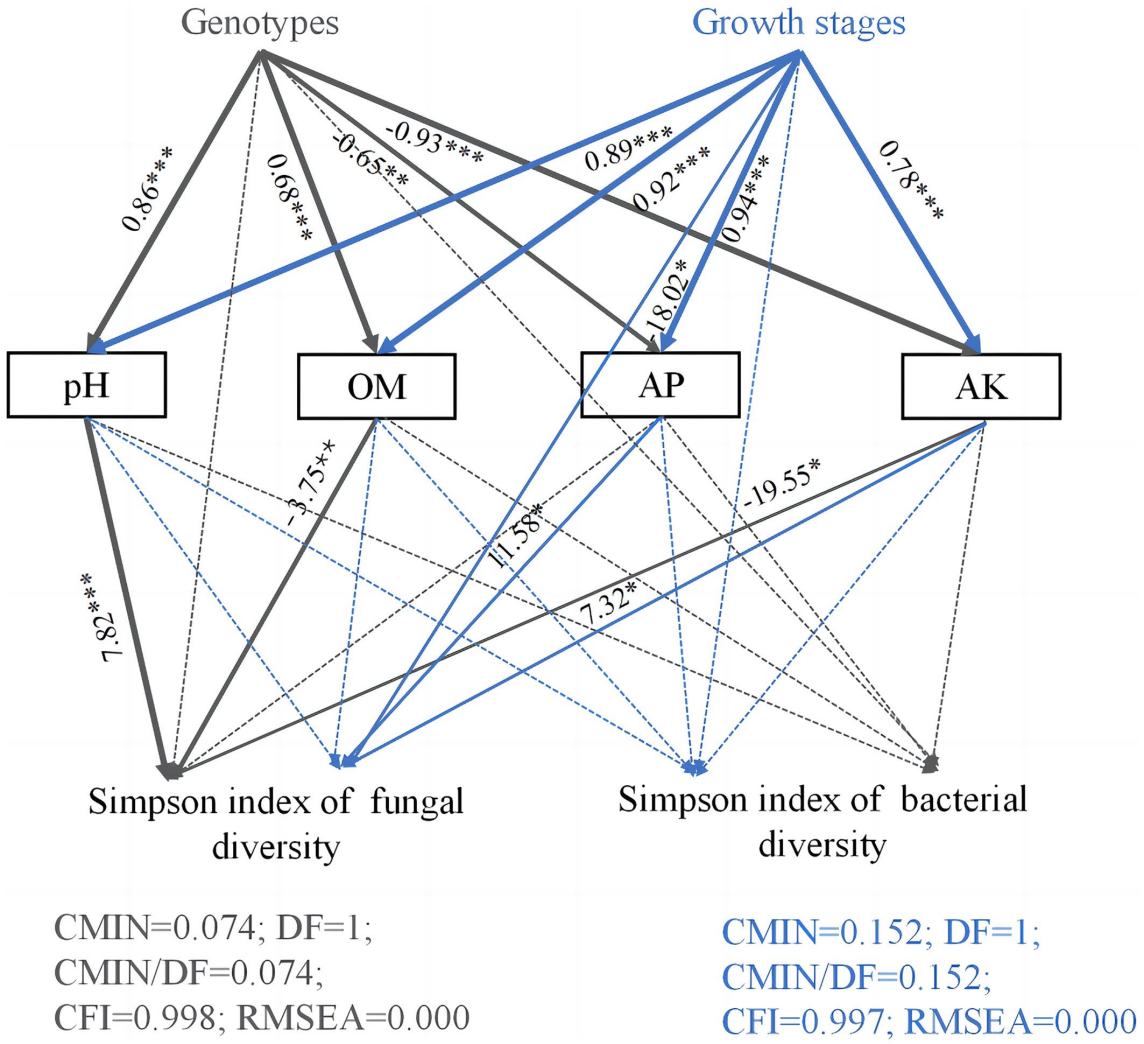
Path diagram of structural equation models (SEMs) showing the effects of genotypes, growth stages, and soil properties on microorganism diversity. Structural equation modeling (SEM) is a method for establishing, estimating, and testing causal relationship models. Path coefficients indicate the magnitude of the relationships between causal factors. The numbers on the lines between the parameter boxes are the standardized regression coefficient. The thickness of the line represents the level of significance (*p < 0.05; **p < 0.01; and ***p < 0.001). The solid and dashed arrows represent the significance and non-significance of the relationship, respectively. OM, organic matter; AP, available phosphorus; AK, available potassium.

Structural equation model path analysis was further used to study the effects of host genotypes, growth stages, and soil chemical properties on Simpson diversity of soil microorganisms in the rhizosphere soils of three *Camellia* genotypes under five plots (Figure **6**). The direct, indirect, and total effects of host genotypes and growth stages on microbial Simpson diversity are shown in Supplementary Table S3. Soil pH, OM, AP, and AK values were significantly affected by host genotypes and growth stages. The fungal Simpson diversity was indirectly influenced by host genotypes, which in turn affected the concentrations of OM, AK, and pH values. In addition, the growth stages had an indirect impact on fungal Simpson diversity through their influence on the concentrations of AP and AK.

## 4 Discussion

The ground diameter and crown width of *C. gauchowensis* were significantly different from those of *C. chekiangoleosa* (Supplementary Figure S2)*. C. chekiangoleosa* is mainly distributed in Zhejiang, Jiangxi, Hunan, and Fujian provinces, with large fruits and leaves, and the ground diameter was significantly smaller than *C. oleifera and C. gauchowensis* (Wang et al., 2022). Significant differences were observed in the chemical properties of rhizosphere soils for the three varieties, which may be associated with the variations in shoot biomass and fine root biomass among different genotypes of *Camellia* (Kong et al., 2020). At the sapling stage, nutrient concentrations in the rhizosphere soil of *C. oleifera* were the lowest. The concentrations of TN, AN, and AK exhibited a trend of initially increasing and then decreasing with the growth stages, which could be attributed to rhizosphere secretions and the accumulation of nutrients in the rhizosphere soil (Zhong et al., 2023).

A strong correlation was found between the properties of rhizosphere soil and microorganisms (Xu et al., 2021). *Mortierella* can promote phosphorus cycling in the rhizosphere (Zhang et al., 2018; Li et al., 2020). *Trichoderma*, a widely utilized biological fungicide in agriculture, primarily functions by triggering plant defense mechanisms and engaging in fungal parasitism (Wang et al., 2020). This study revealed a negative correlation between *Mortierella* and *Trichoderma* with rhizosphere soil pH value, TN, AK, AN, AP, WC, and OM concentrations. Similarly, *Burkholderia–Caballeronia–Paraburkholderia* exhibited a negative correlation with soil chemical properties. Interestingly, it was observed that *Burkholderia–Caballeronia–Paraburkholderia* displayed the ability to colonize and thrive in contaminated soil, suggesting its potential for biological remediation. This could prove advantageous for the growth of *Camellia* roots, particularly under conditions of nutrient deficiencies or other unfavorable circumstances (Mukherjee et al., 2012). *Candidatus Solibacter*, a bacterium capable of decomposing organic matter and utilizing carbon sources (Lin et al., 2022), was also negatively correlated with soil chemical properties. The findings of this study revealed that the growth of *Mortierella*, *Trichoderma*, *Burkholderia–Caballeronia–Paraburkholderia*, and *Candidatus Solibacter* was promoted by low pH and soil nutrient content (Rime et al., 2015). These microorganisms play a beneficial role in nutrient cycling and plant disease suppression. Archaeorhizomycetes play a significant role as a component of the rhizosphere soil microbiota. Previous studies have indicated that their presence reflects the specificity of the ecosystem and the root habitat of the host (Menkis et al., 2014). The species of Archaeorhizomycetes play important roles in the carbon cycle in the surrounding soil of roots (Rosling et al., 2011). This study found a positive correlation between Archaeorhizomyces and the chemical properties of rhizosphere soil, suggesting that improving the chemical properties of rhizosphere soil could enhance soil carbon cycling. By contrast, *Fusarium* is a genus of fungi that includes many species, some of which are pathogenic fungi associated with soil-borne diseases, posing a greater risk to plants with a long growth cycle and impeding plant growth (Li et al., 2019). For instance, *Fusarium oxysporum* can lead to significant losses, including vascular bundle browning, growth retardation, defoliation, and plant death (Rosling et al., 2013).

Research on crops and fruit trees has demonstrated that plant genotypes have an impact on the structure and composition of rhizosphere soil microbial communities (Liu et al., 2022; Dean et al., 2012; Mao et al., 2013). Differences among host genotypes were primarily influenced by rhizosphere soil properties, genetic materials, root morphological structure, and secretions. These factors either promoted or inhibited the growth and development of soil fungi and bacteria, consequently impacting the characteristics of microbial community structure (Kong et al., 2020; Xu et al., 2009; Badri et al., 2013). In addition, the different root-associated microorganisms among plant varieties had different nutritional requirements, which also contributed to the obvious influence of varieties on rhizosphere microbial communities (Cangioli et al., 2022). In this study, Ascomycota and Basidiomycota were the dominant fungal phyla in the rhizosphere soils of *Camellia* forests, which was similar to dominant fungi in the rhizosphere soils of different ecological types from different regions (Li et al., 2021; Dayakar et al., 2009; Egidi et al., 2019). The results were also similar to those obtained by Zhang et al. (2020) in *C. oleifera* forestland (Franke-Whittle et al., 2015). However, the dominant fungi with a relative abundance greater than 1% in rhizosphere soil did not include Zygomycota, which might be linked to significant differences in soil properties. In the soil of *alpine Rhododendron nitidulum* shrubs, the dominant fungal phyla were Ascomycota, Basidiomycota, and Glomeromycotina, which was consistent with the results of this study (Zhang et al., 2020). Basidiomycota, as decomposers of organic matter, play a significant role in nutrient absorption and exchange in plants, thus contributing to afforestation (Li et al., 2021). The relative abundance of Basidiomycota in the rhizosphere soil of *C. gauchowensis* was significantly higher than that of *C. chekiangoleosa* and *C. oleifera* (Figure 3), indicating that *C. gauchowensis* had a greater advantage in the decomposition of dead organic matter and participation in the absorption and transport of root nutrients. This suggests that *C. gauchowensis* may have a more significant impact on nutrient cycling in the soil. In addition, the diversity of *C. gauchowensis* was significantly separated from that of *C. chekiangoleosa* and *C. oleifera*, suggesting that the microbial specificity of *C. gauchowensis* directly impacted the growth and health of *C. oleifera*. It has been identified that *Saitozyma* serves as a key rhizosphere fungal genus in areca nut plants and potentially plays a crucial role in regulating the stability of fungal interaction networks (Xie et al., 2023; Ma et al., 2021). *Mortierella* has the ability to dissolve phosphorus and degrade pesticides to improve the rhizosphere soil and promote plant growth and health (Torres-Cruz et al., 2018). The study results revealed a significantly higher relative abundance of *Saitozyma* and *Mortierella* at the genus level in the G11 rhizosphere soil compared to C11 and H11. This suggests that the symbiotic fungi-associated G11 may have a strong adaptability to environmental limitations, thereby enhancing plant growth.

Proteobacteria, Acidobacteriota, and Chloroflexi were the dominant bacterial phyla in the rhizosphere soils of three *Camellia* genotypes under five plots. This was similar to previous studies on *C. oleifera*, *Angelica sinensis,* and *Aralia continentalis* Kitag (Zhang et al., 2018; Franke-Whittle et al., 2015; Luo et al., 2021). Proteobacteria is the largest phylum of bacteria, and most of them live in the compositive or obligate anaerobic and heterotrophic life with low nutrient requirements. They are widely found in the rhizosphere soils, which are rich in nutrients and suitable for many types of symbiotic bacteria growth including Proteobacteria (Huo et al., 2024). *Burkholderia–Caballeronia–Paraburkholderia* is considered to be a plant growth-promoting rhizobacteria (PGPR) that provides plant hormones and important enzymes for the sugarcane, ginsenosides, and areca nut plants (Xie et al., 2023; Torres-Cruz et al., 2018; Fierer et al., 2007). *Burkholderia–Caballeronia–Paraburkholderia* accounted for a significantly higher proportion in G11, indicating that G11 has better growth performance with the help of this PGPR (Supplementary Figure S2). In addition, Vicinamibacteria are believed to play a role in enhancing phosphate activity and facilitating plant phosphate absorption (Fierer et al., 2007). Thus, *Burkholderia–Caballeronia–Paraburkholderia* and *Vicinamibacterales* can be studied as priority bacterial fertilizers.

Soil microbial communities vary mainly due to soil chemical properties, particularly those related to carbon, nitrogen, and phosphorus nutrient status (Kong et al., 2020; Wu et al., 2022). In this study, the fungal community exhibited a closer network connection at the sapling stage, while displaying a more clustered and closely connected pattern at the primary fruit and full fruiting stages. Mortierellomycota and Chytridiomycota had the largest relative abundance in the rhizosphere soil at the sapling stage of *C. oleifera*, which may be related to the acidic soil pH at the sapling stage (Wang et al., 2019). In addition, *Vicinamibacterales* had positive effects on plant growth, such as enhancing phosphate uptake from soils (Fierer et al., 2007; Jimu et al., 2018; Yu et al., 2022). *Metarhizium* is a diverse genus of fungi well adapted to various ecological niches, such as soil saprotrophs, entomopathogens, and endophytes (Botelho et al., 2019). Within this investigation, the pathogenic fungus *Metarhizium* belongs to entomopathogenic fungi and serves as an excellent biocontrol agent, playing a significant role in promoting plant growth (Sheng et al., 2022). *Metarhizium* is significantly affected by the growth stages of *C. oleifera*, and the proportion of *Metarhizium* increases with the growth stages and is conducive to the healthy growth of *C. oleifera* (Figure 4). Various nematode-pathogenic fungi exist in the genus *Purpureocillium*, such as *Purpureocillium lilacinum*, a soil fungus widely tested for biological control of plant-parasitic nematodes. Khan and Tanaka (2023) were able to show that *P. lilacinum* could significantly increase the biomass and photosynthesis of nematode-infected carnation plants, thereby promoting their growth. A recent study demonstrated that *Schizothecium* serves as a biocontrol agent against soil-borne pathogens, with its abundance strongly correlated to soil nitrogen content (Lv et al., 2022). The relative abundance of *Schizothecium* in the rhizosphere soil of *C. oleifera* was significantly higher at the fruiting stage compared to the sapling and primary fruit stages. This increase may be linked to the soil fertility during the plant’s growth stages. Therefore, soil nutrient concentrations should be ensured at the primary fruit stage of *C. oleifera* to enrich the beneficial Basidiomycota, which is conducive to the formation of mutualistic relationships between fungi and plants. During the full fruiting stage, it is important to control pathogenic fungi such as *Metarhizium* to reduce the risk of plant disease. In addition, applying beneficial fungi such as *Schizothecium* can help improve disease resistance. In this study, we did not analyze the microorganisms in the collected soil to verify their effects on *C. oleifera* plants. In addition, we did not investigate the function of the microorganisms through molecular biology and microbiology. The sample of this study only studied the oil tea forests in Xiaokeng Forestry in South China, so the data obtained in this study may have some limitations, and relevant studies in different areas are needed in subsequent studies. In addition, our study addressed three different *Camellia* species at three different ages (*C. oleifera*—at age 4, 7, and 11, *C. gauchowensis*—at age 11, and *C. chekiangoleosa*—at age 11). While comparisons across the different ages of *C. oleifera* and the three different species at age 11 provided valuable insights, the limitations and challenges especially due to the imbalance while sampling across ages are noted. Therefore, this study will follow up by exploring both of these aspects. In addition, we plan to isolate and identify the beneficial microbes abundant in the rhizosphere soils of *Camellia* plants. Subsequently, we aim to inoculate these beneficial microbes into *Camellia* plants to investigate their effects on plant growth and development.

## 5 Conclusion

In conclusion, the diversity and composition of microbiota in rhizosphere soils of *Camellia* forests were closely related to the soil organic matter (OM) and water content (WC). They were significantly affected by host genotypes and growth stages. Fungal diversity was more sensitive to the growth stage, and microbial diversity was similarly affected by genotype. During the early fruit stage of *C. oleifera* plants, it is important to inoculate beneficial microorganisms such as *Burkholderia–Caballeronia–Paraburkholderia*, *Vicinamibacterales*, *Schizothecium*, and *P. lilacinum*. This inoculation strategy fosters mutualistic relationships between microorganisms and plants, ultimately leading to improved plant health and productivity. Soil pH value and the soil OM, WC, TN, AN, and AK concentrations were the key factors that affected the microbial community in the rhizosphere soils of *Camellia*. The improvement of soil chemical properties can shape the root microbiota and affect nutrient circulation and substance transformation, thereby contributing to the adjustment of the rhizosphere microenvironment and enhancing the growth and health of forest trees.

## Data Availability

The datasets presented in this study can be found in online repositories. The names of the repository/repositories and accession number(s) can be found at: https://www.ncbi.nlm.nih.gov/; https://www.ncbi.nlm.nih.gov/sra/PRJNA910656; https://www.ncbi.nlm.nih.gov/; https://www.ncbi.nlm.nih.gov/sra/PRJNA910505.
