# Supplementary material for: Host genotype and age shape the microbial community in the rhizosphere soils of Camellia forests
Source: Front Microbiol. 2024 Oct 1;15:1440255. doi: 10.3389/fmicb.2024.1440255 (PMC11477377; doi:10.3389/fmicb.2024.1440255)
Supplement: Supplementary file 1 [file Data_Sheet_1.docx]

Supplementary Figures:


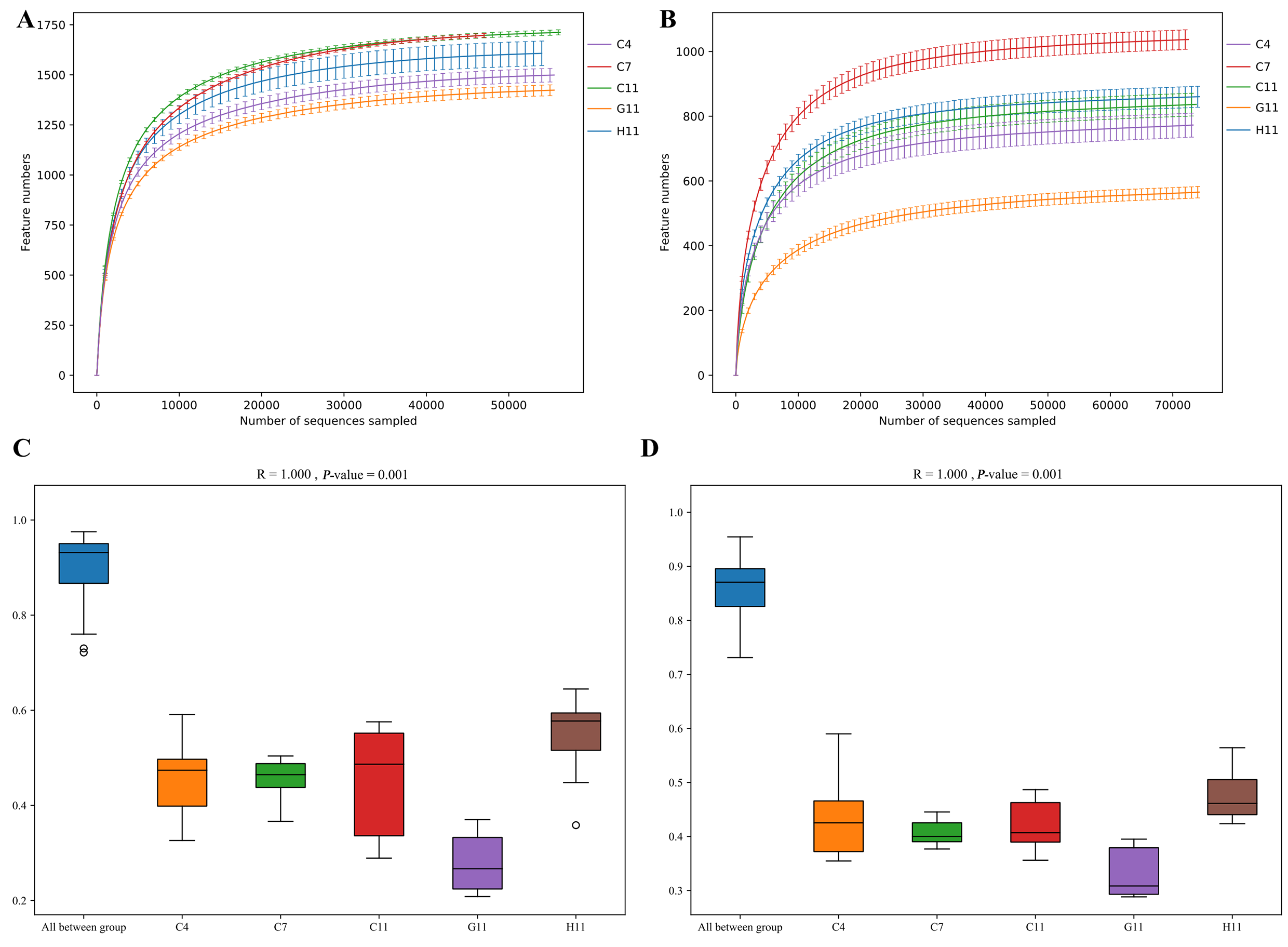


Figure S1. Rarefaction curves and alpha diversity indexes of microbial community in the rhizosphere soils of three *Camellia* genotypes under five plots. (A) Rarefaction curves of fungal communities. (B) Rarefaction curves of bacterial communities. ANOSIM based on the Bray–Cutis of fungal (C) and bacterial (D) community among 25 samples in the rhizospheric soil of *Camillia*. The same below. *R* > 0 indicates significant component differences. *P*<0.05 indicates statistical significance.C4: sapling stage of *C. oleifera*; C7: primary fruit stage of *C. oleifera*; C11: full fruiting stage of *C. oleifera*; G11: full fruiting stage of *C. gauchowensis*; H11: full fruiting stage of *C. chekiangoleosa*.


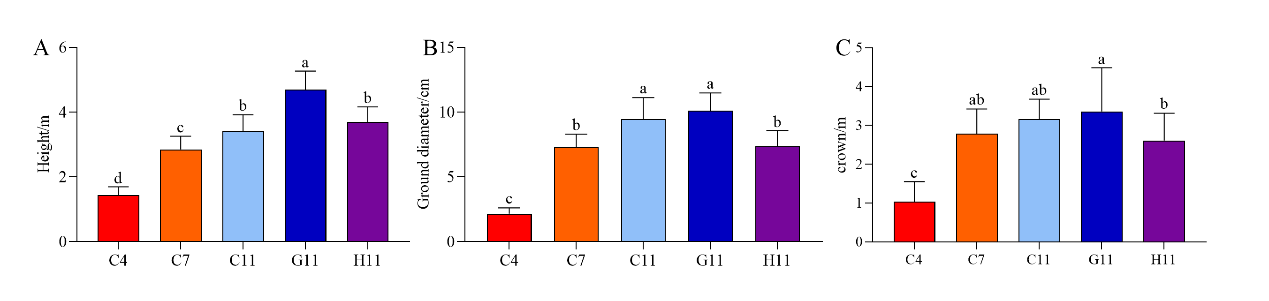


Figure S2. The height, ground diameter and crown width of three *Camellia* genotypes under five plots. Different letters indicated significant differences in individual parameters among five plots by the Duncan’s test (*P* < 0.05). C4: sapling stage of *C. oleifera*; C7: primary fruit stage of *C. oleifera*; C11: full fruiting stage of *C. oleifera*; G11: full fruiting stage of *C. gauchowensis*; H11: full fruiting stage of *C. chekiangoleosa*.

Table S1. Characteristics of three genotypes of *C. oleifera*

| Variety | Age | Distribution region | Fruit color | Fruit size/mm | Blooming stage |
| --- | --- | --- | --- | --- | --- |
| *C. oleifera* | 4&7&11 | Widely distributed | Cyan yellow | 60.62×47.17 | mid November -late December |
| *C. gauchowensis* | 11 | Guangdong, Hainan, Vietnam | Cyan yellow | 69.46×77.22 | Early November -late January |
| *C. chekiangoleosa* | 11 | Zhejiang, Jiangxi, Hunan and Fujian Province | Red | 50.0×70.0 | February -march |


Table S2. Correlation analysis between top ten dominant microbial genus and environmental factors in rhizosphere soil of three *Camellia* genotypes under five plots.

| Environmental factors | Fungi | | | | Bacteria | | | |
| --- | --- | --- | --- | --- | --- | --- | --- | --- |
|  | RDA1 | RDA2 | R^2^ | *P*-value | RDA1 | RDA2 | R^2^ | *P*-value |
| pH | -0.99808 | -0.06192 | 0.361146 | 0.074 | -0.98219 | 0.187882 | 0.726061 | 0.002 |
| OM | -0.69699 | 0.717078 | 0.829432 | 0.001 | -0.81817 | -0.57498 | 0.559871 | 0.01 |
| TN | -0.88019 | 0.474622 | 0.815565 | 0.001 | -0.75227 | -0.65885 | 0.789308 | 0.001 |
| AN | -0.89024 | 0.455495 | 0.669839 | 0.001 | -0.59881 | -0.80089 | 0.738227 | 0.001 |
| AP | 0.071292 | 0.997455 | 0.07016 | 0.62 | 0.024899 | 0.99969 | 0.045975 | 0.73 |
| AK | -0.95606 | -0.29317 | 0.479011 | 0.023 | -0.99947 | 0.032465 | 0.600029 | 0.007 |
| WC | -0.9243 | 0.381658 | 0.860164 | 0.001 | -0.86565 | -0.50065 | 0.908983 | 0.001 |

* The *P***-**values of RDA1 and RDA2 were the cosine of the angle between the soil environmental factor arrow and the sorting axis; R^2^ represented the determination coefficient of environmental factors on genus distribution. OM: Organic matter; TN: Total nitrogen; TK: Total potassium; AN: Alkali hydrolyzed nitrogen; AP: Available phosphorus; AK: Available potassium; WC: water content.

Table S3. Network map connectivity statistical in rhizosphere soil microbial community of three *Camellia* genotypes under five plots.

| Network map | Fungi | | | | | Bacteria | | | | | |
| --- | --- | --- | --- | --- | --- | --- | --- | --- | --- | --- | --- |
|  | A | B | C | D | E | | F | G | H | I | J |
| nodes num | 79 | 81 | 76 | 72 | 72 | | 54 | 84 | 56 | 79 | 63 |
| edges num | 100 | 100 | 100 | 100 | 100 | | 100 | 100 | 100 | 100 | 100 |
| average degree | 2.53 | 2.47 | 2.63 | 2.78 | 2.78 | | 3.70 | 2.38 | 3.57 | 2.53 | 3.17 |
| average path length | 1.09 | 1.44 | 2.40 | 1.22 | 1.15 | | 1.35 | 1.25 | 1.16 | 1.07 | 1.34 |
| graph diameter | 2 | 6 | 5 | 3 | 3 | | 2 | 3 | 2 | 2 | 2 |
| graph density | 0.03 | 0.03 | 0.04 | 0.04 | 0.04 | | 0.07 | 0.03 | 0.06 | 0.03 | 0.05 |
| average clustering coefficient | 0.91 | 0.86 | 0.81 | 0.89 | 0.92 | | 0.74 | 0.98 | 0.86 | 0.94 | 0.71 |
| modularity | 0.90 | 0.92 | 0.88 | 0.87 | 0.79 | | 0.74 | 0.92 | 0.81 | 0.92 | 0.66 |
